# Comment on: Routine practice needs shifting from linear to purse-string skin closure in closure of stoma sites

**DOI:** 10.1007/s00384-020-03560-8

**Published:** 2020-03-17

**Authors:** Philipp-Alexander Neumann, Stefan Reischl, Helmut Friess, Daniel Reim

**Affiliations:** Department of Surgery, Klinikum rechts der Isar, School of Medicine, Technical University of Munich, Ismaninger Str. 22, 81675 Munich, Germany

Dear Editor:

We thank Hajibandeh et al. for their interest in our work and their comment [1] related to our recent publication “Meta-analysis and single-center experience on the protective effect of negative suction drains on wound healing after stoma reversal.” [2]

Regarding their remark in respect to the differences in wound healing complications following purse string vs. primary closure, we have to state that this comparison was not part of our analysis. In our opinion, it is difficult to assess wound infection rates in purse-string closure, since purse string is intended to heal in secondary intention. We do see the advantages of the smaller incision and therefore minimized scarring following purse-string closure. However, as stated in our article, purse-string closure is not possible in all cases and also has its specific disadvantages like the prolonged time to secondary healing especially in obese patients.

Therefore, we conducted our analysis to see whether negative suction drainages still have their place in reducing wound complications in wounds that are at risk following ileostomy reversal [2]. As we have shown, use of negative suction drainages can significantly reduce wound complications. However, as shown in our data, the complication rate remains considerably high and still higher than what has been reported for the purse-string technique. We therefore conclude that, whenever possible, purse string might be the better option. If it is not possible to use purse string (obese patients, extended resections with widening of the incision), we recommend the use of a negative suction drain for reduction of complications.
